# Metastatic Large Cell Neuroendocrine Carcinoma of the Colon: A Case Report

**DOI:** 10.7759/cureus.26075

**Published:** 2022-06-19

**Authors:** Vatsal Khanna, Trishya Reddy, Tripti Nagar, Vesna Tegeltija

**Affiliations:** 1 Department of Internal Medicine, Wayne State University School of Medicine, Rochester Hills, USA

**Keywords:** immunohistochemistry, metastatic, adenocarcinomas, colorectal, neuroendocrine

## Abstract

Colorectal neuroendocrine tumors are rare entities, with large cell neuroendocrine carcinomas occurring less frequently. We report a case of an 83-year-old male who presented with symptoms of intestinal obstruction. A computed tomography scan of the abdomen and pelvis revealed a high-grade large bowel obstruction secondary to an irregular exophytic soft tissue mass within the ascending colon, with extensive metastatic disease. He subsequently underwent a right hemicolectomy. Histologic evaluation revealed large cell neuroendocrine carcinoma of the colon. Standardized treatment modalities have not been established; however, chemotherapy is often used as the first-line or adjuvant therapy with surgery. Unfortunately, our patient succumbed to postoperative complications on day 30 of the hospital stay.

## Introduction

Neuroendocrine neoplasms (NENs) can occur anywhere in the body, more commonly occurring in the lung and gastroenteropancreatic (GEP) regions. Among the GEP tumors, colorectal neuroendocrine tumors are rare and mostly carcinoid. Neuroendocrine carcinomas are a subtype of NENs and can be classified into two types: small cell and large cell carcinomas. 

Large cell neuroendocrine carcinomas (LCNECs) are rare tumors making up less than 1% of colorectal carcinomas, with a more aggressive clinical course and poorer prognosis than adenocarcinomas [1]. LCNECs have characteristic histological features such as (i) organoid, nesting, trabecular, rosette, and palisading patterns, (ii) large cells with a polygonal shape, coarse chromatin, and frequent nucleoli, and (iii) very high mitotic rate along with frequent areas of necrosis under light microscopy. The diagnosis is confirmed by immunohistochemical staining. 

Surgical resection of the tumor is the preferred first-line treatment to relieve the obstruction; however, no consensus exists for treating colorectal neuroendocrine carcinomas [2]. We report a case of an 83-year-old male who presented to our hospital with a large bowel obstruction and was found to have LCNEC on immunohistochemistry.

## Case presentation

We report a case of an 83-year-old male with multiple comorbidities who was admitted to the hospital with complaints of abdominal pain for the past two days, which was insidious in onset, diffuse, colicky, and non-radiating, associated with nausea, bilious vomiting, and constipation for one week. There were no aggravating or relieving factors. The patient denied unintentional weight loss, fatigue, melena, or hematochezia. The patient states his last colonoscopy was ten years ago and was normal. His family history was negative for colorectal malignancy.

**Figure 1:**
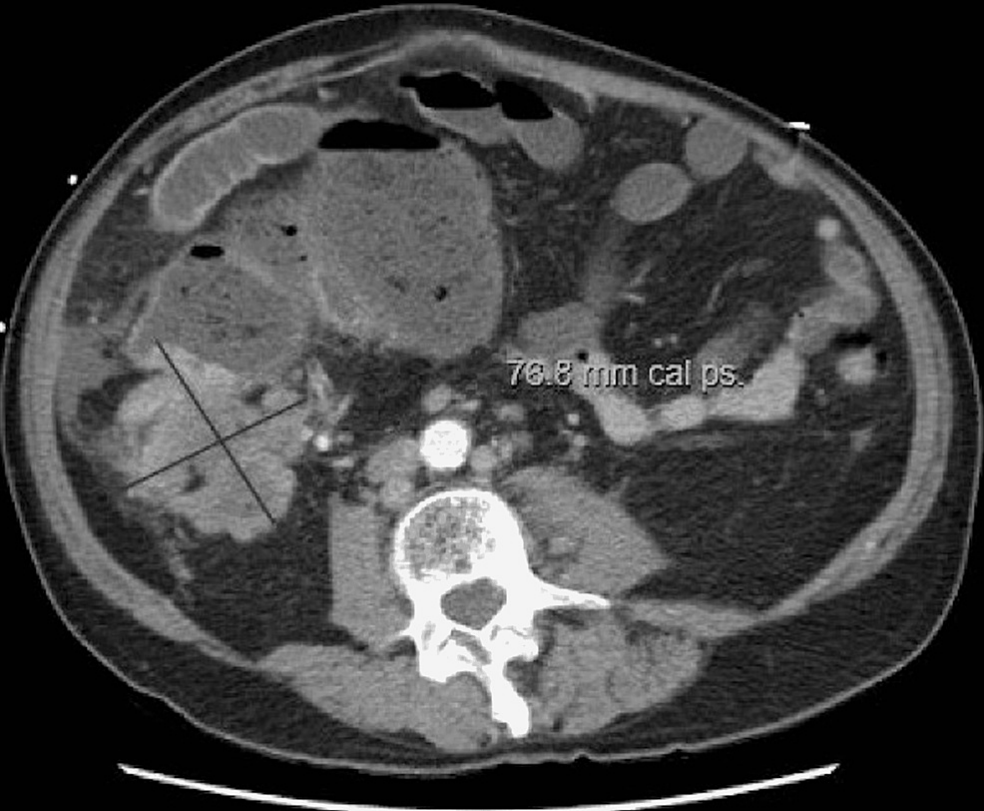
CT abdomen and pelvis showing irregular exophytic soft tissue mass within the ascending colon, with enlarged retroperitoneal lymph nodes

On admission, the patient was normotensive, heart rate was 110 bpm, afebrile, and saturated 98% on room air. Clinical laboratory results at the time of admission were unremarkable. Physical examination revealed a distended abdomen with diffuse lower abdominal tenderness and no signs of peritonitis. A computed tomography (CT) scan of the abdomen and pelvis with intravenous and oral contrast was obtained, which showed a high-grade large bowel obstruction secondary to an irregular exophytic soft tissue mass within the ascending colon, with extensive metastatic disease to the liver with abdominal lymphadenopathy (Figure 1). Gastroenterology and general surgery were consulted. Given the severity of obstructive symptoms, the patient was taken up for emergent exploratory laparotomy and underwent a right hemicolectomy.

**Figure 2:**
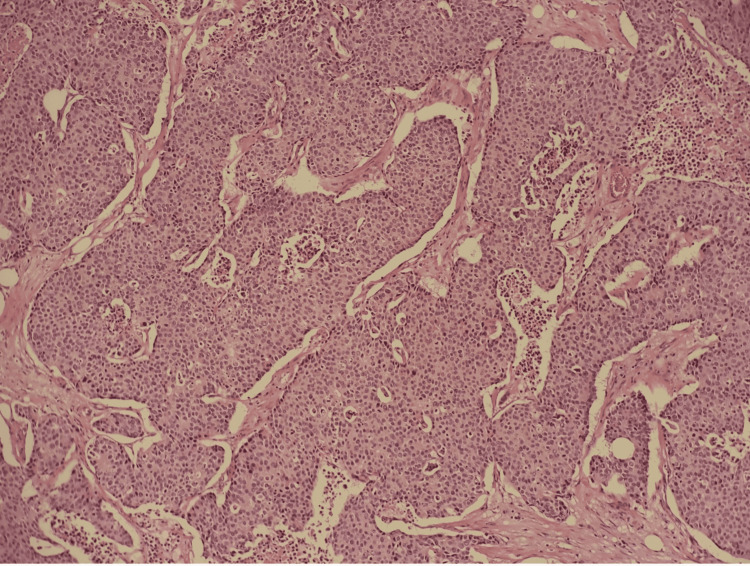
Solid nests typical of neuroendocrine neoplasms on hematoxylin and eosin staining (magnification 100x)

**Figure 3:**
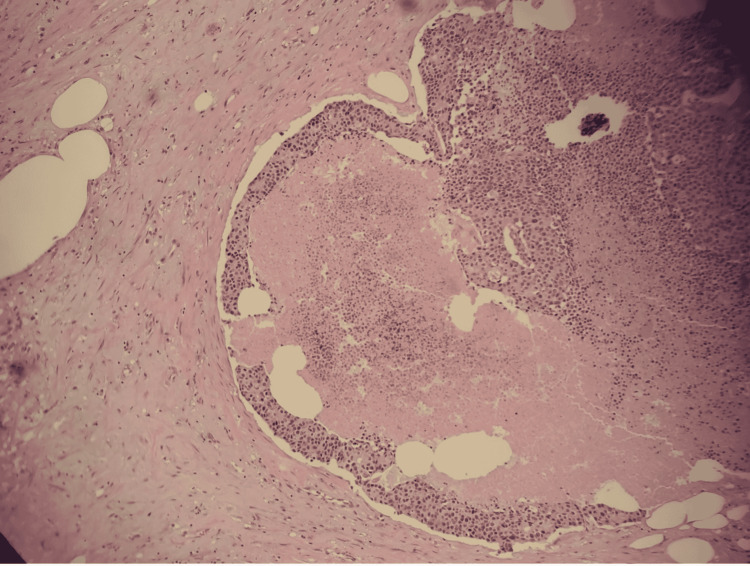
Geographic areas of necrosis suggestive of poor differentiation on hematoxylin and eosin stain (magnification 100x)

**Figure 4:**
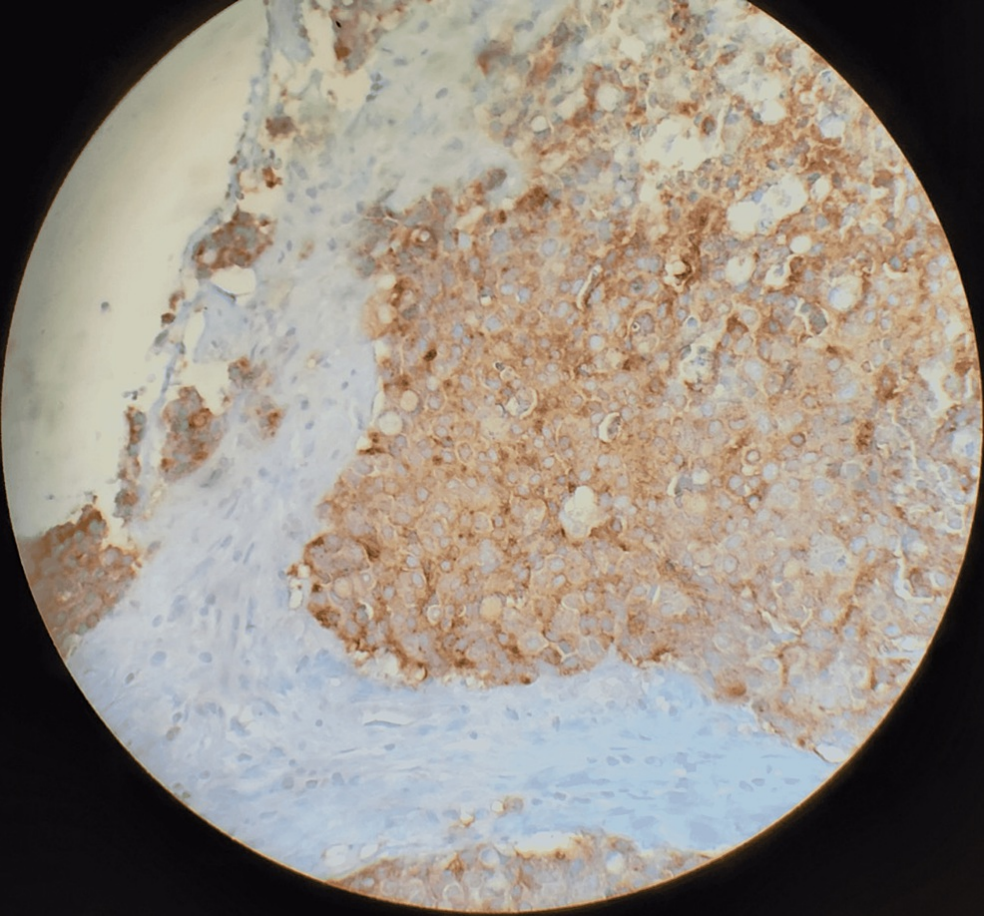
Tumor showing diffusely positive synaptophysin expression (magnification 100x)

Histopathologic examination revealed solid nests of poorly differentiated neuroendocrine carcinoma of the ascending colon invading the muscularis propria into pericolic tissues with a high mitotic rate and evidence of angiolymphatic invasion pTNM staging: T3 N1 M1b grade 3 neuroendocrine tumor (Figures 2, 3). In addition, the immunohistochemical staining was diffusely positive for CK20, CDX2, and synaptophysin, confirmatory of LCNEC, while negative for CK7 and chromogranin (Figure 4).

The patient’s postoperative period was complicated by septic shock secondary to fecal peritonitis from an anastomotic leak. As a result, he developed multiorgan failure and eventually succumbed to these complications on day 30 of his hospital stay.

## Discussion

Neuroendocrine cells are present throughout the length of the gut, pancreas, and lung, with neuroendocrine tumors (NET) commonly arising in these areas. NETs in the colon are rare; most of these are carcinoid tumors with a relatively good prognosis. However, a small number are LCNECs. A study published by Bernick et al. showed that NETs made up 0.6% of malignant colorectal tumors, and only 0.2% of those were large cell neuroendocrine carcinomas [1].

LCNECs can mimic colonic adenocarcinomas in their clinical presentation. Symptoms include abdominal pain, hematochezia, tenesmus, and obstipation. As in our case, the patient presented with obstruction and abdominal pain, with no complaints associated with hormone overproduction, which can be seen with carcinoid tumors [2]. LCNECs typically have a higher grade of metastasis at presentation, making the prognosis very poor, with studies showing a median survival time of five to 11 months and a one-year survival rate of 10% [3]. The CT scan of the abdomen in our patient showed multiple hepatic masses, the largest measuring 4.2 cm in the right hepatic lobe, consistent with metastases and enlarged retroperitoneal and upper abdominal lymph nodes.

The novel WHO classification published in 2010 uses the Ki-67 index, the marker for cell proliferation, and mitotic count to classify NETs into well-differentiated tumors (G1 and G2) or poorly differentiated tumors, now called large cell or small cell type G3 neuroendocrine carcinomas. G1 comprises well-differentiated NETs that are low grade, with a low mitotic rate of < 2 per 10 high-power fields (HPF) and a Ki-67 index of < 2%. G2 comprises well-differentiated NETs that are intermediate grade, with a mitotic rate of 2-20 per 10 HPF and a Ki-67 index of 3-20%. G3 NETs are poorly differentiated, with a high mitotic rate of >20 per 10 HPF and a Ki-67 index of >20% [4].

Common histopathological findings of LCNECs are organoid patterns with solid nests, rosette formation, focal necrosis, and a high mitotic rate. Synaptophysin is diffusely positive in almost all NECs, while chromogranin A can be frequently negative. LCNECs often resemble poorly differentiated adenocarcinomas, making testing for neuroendocrine markers important [5]. The tumor in our case showed solid nests of poorly differentiated LCNEC with a high mitotic rate and areas of necrosis (Figure 3). It was diffusely positive for CK20, CDX2, and synaptophysin, while negative for CK7 and chromogranin.

Unlike adenocarcinomas, most poorly differentiated LCNECs are negative for CK-20 (a tumor marker typically found in intestinal epithelial, urothelial, and Merkel cells). However, Kato et al. reported a case of CK-20 positive LCNEC suggesting a potential link between these tumors and conventional colonic adenocarcinoma [6].

Currently, there are no standardized treatment guidelines for colorectal LCNECs. Radical excision is a treatment option for local, non-metastatic disease. Chemotherapy with a platinum-based regimen is frequently used as first-line treatment in unresectable tumors or as adjuvant therapy to surgery [5,7]. Patients with metastatic disease may benefit from surgical interventions to deal with tumor complications such as obstruction, bleeding, and perforation, as in our case [7]. However, a retrospective review conducted by Smith et al. found that surgery in the presence of metastatic disease may not offer a survival benefit for most of these patients [8]. 

## Conclusions

Large cell neuroendocrine carcinomas of the colon are rare tumors with a poor prognosis due to their aggressive nature. They have a median survival rate of less than a year. Therefore, early detection plays a vital role in the management of these patients, as these aggressive tumors metastasize rapidly. 

Pathologically, these tumors are poorly differentiated carcinomas with distinctive cytoarchitectural features and are often immunoreactive for markers of neuroendocrine differentiation. Tumors diagnosed as poorly differentiated carcinomas by biopsy should be treated considering neuroendocrine carcinomas as a differential diagnosis. Further research is needed to establish standardized treatment modalities, particularly adjuvant chemotherapy regimens.
